# A Reanalysis of Cognitive-Functional Performance in Older Adults: Investigating the Interaction Between Normal Aging, Mild Cognitive Impairment, Mild Alzheimer's Disease Dementia, and Depression

**DOI:** 10.3389/fpsyg.2015.02061

**Published:** 2016-01-26

**Authors:** Jonas J. de Paula, Maria A. Bicalho, Rafaela T. Ávila, Marco T. G. Cintra, Breno S. Diniz, Marco A. Romano-Silva, Leandro F. Malloy-Diniz

**Affiliations:** ^1^Faculdade de Medicina, Instituto Nacional de Ciência e Tecnologia de Medicina Molecular, Universidade Federal de Minas GeraisBelo Horizonte, Brazil; ^2^Department of Psychology, Faculdade de Ciências Médicas de Minas GeraisBelo Horizonte, Brazil; ^3^Department of Internal Medicine, Faculdade de Medicina, Universidade Federal de Minas GeraisBelo Horizonte, Brazil; ^4^Reference Center for Geriatrics Instituto Jenny de Andrade Faria, Universidade Federal de Minas GeraisBelo Horizonte, Brazil; ^5^Department of Mental Health, Faculdade de Medicina, Universidade Federal de Minas GeraisBelo Horizonte, Brazil

**Keywords:** older adults, depression, cognitive assessment, activities of daily living, neuropsychological assessment, mild cognitive impairment, Alzheimer's disease

## Abstract

Depressive symptoms are associated with cognitive-functional impairment in normal aging older adults (NA). However, less is known about this effect on people with mild Cognitive Impairment (MCI) and mild Alzheimer's disease dementia (AD). We investigated this relationship along with the NA-MCI-AD continuum by reanalyzing a previously published dataset. Participants (*N* = 274) underwent comprehensive neuropsychological assessment including measures of Executive Function, Language/Semantic Memory, Episodic Memory, Visuospatial Abilities, Activities of Daily Living (ADL), and the Geriatric Depression Scale. MANOVA, logistic regression and chi-square tests were performed to assess the association between depression and cognitive-functional performance in each group. In the NA group, depressed participants had a lower performance compared to non-depressed participants in all cognitive and functional domains. However, the same pattern was not observed in the MCI group or in AD. The results suggest a progressive loss of association between depression and worse cognitive-functional performance along the NA-MCI-AD continuum.

## Introduction

Depressive symptoms and cognitive impairment are common in older adults and often coexist in an individual patient. The pattern of cognitive impairment associated with depressive symptoms involves executive dysfunction, reduced processing speed, and deficits in episodic memory (Butters et al., 2004; Sexton et al., 2012; de Paula et al., 2013a), while global intellectual ability, language skills, visuospatial abilities, and semantic processing are usually spared (Naismith et al., 2003). Furthermore, late-life depression is a risk factor for cognitive decline and dementia, in particular Alzheimer's disease dementia (AD) and vascular dementia (Diniz et al., 2013).

The neurobiological mechanisms connecting the depressive symptoms with cognitive and functional performance are heterogeneous. Naismith et al. (2012) reviewed evidences from different types of studies, which discussed changes in monoamine systems dysfunction, hormonal and immunologic changes, inflammatory processes, and alterations on genes expression. These different mechanisms may increase neurodegenerative and vascular factors, which may mediate the cognitive and functional changes associated with depression (Butters et al., 2008). However, as reviewed by Panza et al. (2010), there is an important overlap between depression, mild cognitive impairment (MCI) and dementia: depression may overlap with clinical MCI, may be a reaction to the initial symptoms of MCI, may be one of the behavioral manifestations of MCI, or may mask a clinical MCI. As reviewed by the authors, differences in the studies settings and design, participants' characteristics, and the procedures to diagnose depression and MCI may contribute to the inconsistency found among the studies, including the prevalence of these conditions, its cognitive features and risk of conversion to dementia.

Despite the well-documented impact of depressive symptoms on cognition, the relationship of symptoms' intensity and cognitive performance on older adults with neurocognitive disorders, in particular MCI and AD, is controversial. Previous studies found no association between depressive symptoms and neuropsychological performance in mild AD or MCI patients. Bangen et al. (2010) assessed depressed and non-depressed AD patients with a comprehensive neuropsychological battery and found no differences on the cognitive performance of these participants. On the other hand, AD patients with depression showed a greater impairment in attention and executive function tests when compared to non-depressed AD patients in another study (Nakaaki et al., 2007). A recent study showed that depressive symptoms are related to the cognitive decline in AD, even after controlling for baseline cognitive status (Zahodne et al., 2013). The controversy occurs on the relationship between depression and functional performance. Depressive symptoms are associated with poorer performance on activities of daily living (ADL) in community-dwelling and institutionalized older adults (Nyunt et al., 2012; Tomita and Burns, 2013; de Paula et al., 2015a), and are important predictors of functional status in MCI subjects (Bombin et al., 2012). The presence of cognitive impairment and comorbid depressive symptoms seems to lead to worse functional outcomes (Wadsworth et al., 2012). On the other hand, different studies did not find a significant association between depressive symptoms and functional status along the normal aging (NA)—MCI—AD continuum (Reppermund et al., 2011).

Altogether, these results suggest that depressive symptoms may have a distinct impact on cognitive and functional performance in normal aging, MCI and AD. In this sense, the aim of the present study is to evaluate how depressive symptoms moderate the cognitive and functional performance along this continuum. We have reanalyzed a dataset used in previous studies (de Paula et al., 2013b, 2014, 2015a).

## Materials and methods

### Participants

We included 274 older adults in the present study from a convenience sample. This study was approved by the local ethics board (registry 334/06). All subjects gave written informed consent for participation. The caregivers of dementia patients had also filled the consent form. The study is in accordance with the Declaration of Helsinki. These participants and their data were described in previously published studies (de Paula et al., 2013b, 2014, 2015a). Participants were evaluated at the outpatient geriatric clinic in the Federal University of Minas Gerais, Brazil. Patients which in primary-care medical assessment had memory complains or voluntarily asked for a cognitive assessment were referred for the outpatient clinic, where a comprehensive assessment was performed and they were subsequently invited for participation. Most of these patients has a very low formal education (below 5 years) and low socioeconomic status.

**Figure 1:**
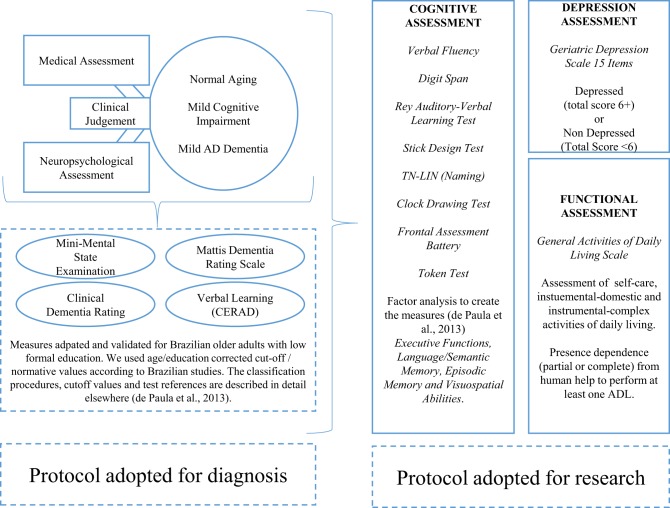
**Description of the protocol used for participant's diagnosis/classification, and procedures adopted for this study**. Participants initially underwent a medical and neuropsychological assessment for diagnostic procedures. In all, 274 older adults were divided into Normal Aging (NA, *N* = 96), mild cognitive impairment (MCI, *N* = 85), mild dementia, and Alzheimer's disease dementia (AD, *N* = 93). After diagnosis, they performed the procedures of the study, involving the assessment of cognitive functioning, depression and activities of daily living.

Figure 1 shows the methods used for patient's diagnoses and the methods used for this research. The Brazilian version of the Mattis Dementia Rating Scale (Porto et al., 2003), the Mini-Mental State Examination (MMSE; Folstein et al., 2001), subtests of the CERAD Neuropsychological Battery (Morris et al., 1989), and the Clinical Dementia Rating (CDR; Morris, 1993) were adopted. CDR scores below 1 are not indicative of dementia, while those equal or above this value are indicative of mild (1), moderate (2), or severe (3) dementia (Morris, 1993). Only participants with CDR scores equal or below 1 were included. The performance on cognitive tests was adjusted for age and educational status, based on Brazilian norms, and were previously validated for the diagnosis of mild AD and MCI (Brucki et al., 2003; Porto et al., 2003; Nitrini et al., 2004; Foss et al., 2013; Table 1 shows the participants sociodemographic characteristics, performance in cognitive tests used for diagnosis and the median for each test based in normative data).

**Table 1:** **Participant's description in sociodemographic and cognitive measures used for diagnosis**.

**Measures**		**Normative data^a^**	**NA**		**MCI**		**AD**	
		**(median)**	***M***	***SD***	***M***	***SD***	***M***	***SD***
Sociodemographic	Age	–	72.61	7.76	73.18	8.46	74.57	6.65
	Education	–	5.22	4.29	4.71	4.00	4.82	3.46
Global measures	MMSE	26	25.75	3.85	23.52	3.62	20.59	3.98
	DRS—Total	129	128.02	9.24	114.56	12.93	97.36	12.20
Attention	DRS—Attention	35	35.34	1.41	34.71	1.75	33.61	2.17
Executive functions	DRS—Initiative/Perseveration	33	32.92	4.46	28.32	5.66	22.82	5.40
Visuospatial abilities	DRS—Construction	6	5.42	1.13	5.25	3.50	4.14	1.89
Language	DRS—Conceptualization	32	32.26	4.92	28.48	6.46	24.23	5.26
Memory	DRS—Memory	23	22.04	2.44	18.06	3.77	12.55	3.99
	CERAD-VL (Total)	18	16.58	2.3	12.87	2.14	10.44	2.15
	CERAD-VL (Recall)	5	5.49	1.83	2.18	1.63	2.11	1.79
	CERAD-VL (Recognition)	9	8.89	1.41	6.69	1.69	3.44	2.17

*NA, Normal Aging; MCI, Mild Cognitive Impairment; AD, Alzheimer's disease dementia; MMSE, Mini-Mental State Examination; DRS, Mattis Dementia Rating Scale; CERAD-VL, Verbal Learning Test from The Consortium to Establish a Registry for Alzheimer's disease neuropsychological battery*.

a *Based in Brazilian normative/adaptation studies with older adults with similar age (±75 years) and education (±4 years)*.

Cognitive status was adjudicated in expert multidisciplinary meetings taking into account clinical, cognitive assessment, laboratorial, and neuroimaging data when available. We performed the diagnosis of probable AD according to the NINCDS-ADRDA criteria (McKhann et al., 1984). It involved cognitive impairment objectively assessed by neuropsychological tests of episodic memory and at least on other cognitive domain (Executive Functions, Visuospatial Abilities, and Language/Semantic Memory) from subtests of the Brazilian versions of the CERAD Neuropsychological Battery and the Mattis Dementia Rating Scale; functional impairment when compared to a previous level of performance (assessed by the functional components of the CDR and by a clinical interview conducted by an experienced geriatrician); insidious onset; clear-cut report of cognitive/functional decline; and no evidence of major neurologic or psychiatric conditions which could respond for the symptom.

The diagnosis of MCI was done according to the following criteria, adapted from Petersen et al. (2001): (1) subjective cognitive complaint, preferably corroborated by an informant; (2) objective impairment in the performance on specific cognitive tests of the assessment battery (verbal learning test from the CERAD battery and subscales from the Mattis Dementia Rating Scale); (3) preserved global cognitive functioning [MMSE above the cut-off for dementia adjusted for education (as in Brucki et al., 2003), and CDR < 1]; (4) preserved or minimal impairments in ADL assessed by a clinical interview and the functional components of the CDR; (5) not demented. The MCI patients included on the study has symptoms and progression characteristic of Alzheimer's disease (amnestic presentation—deficits in learning and recall of new information, insidious onset, and clear-cut history of worsening of cognition by caregiver report).

We also assessed older adults with no evidence of cognitive impairment in the screening protocol to form a control group without clinical history suggestive of Alzheimer's disease. These participants composed the normal aging group (NA). We invited them for participation in the same institution where we assessed the MCI and AD participants. They do not show a CDR suggestive of dementia (CDR < 1) and has cognitive tests above the cut-off values for cognitive impairment. Participants with subjective cognitive complaints did not have a symptomatology or clinical course suggestive of Alzheimer's disease (as cited in AD and MCI participant's description). These participants have none or discrete functional impairment assessed in a clinical interview, no history of neurological diseases and no sensorial or motor impairment, which may compromise the neuropsychological assessment.

Our final sample consisted of 274 participants: 62 NA non-depressed, 34 NA depressed 63 MCI non-depressed, 22 MCI depressed, 66 AD non-depressed, and 27 AD depressed. We described these previous in previously published studies (de Paula et al., 2013b, 2014, 2015a).

### Assessment of depressive symptoms

We evaluated the presence of depression by the Brazilian version of the Geriatric Depression Scale-15 (GDS-15; Sheikh and Yeasavage, 1986). The cut-off score 5/6 (non-case/case) was chosen to determine the presence of clinically significant depressive. We adopted this cut-off due to its good validity for detection of significant depressive symptoms in our population, which includes patients with low socioeconomic status, low formal education, and cognitive complaints (Almeida and Almeida, 1999). A validation study propose this cut-off for the detection of depression according to the DSM-IV and ICD-10 criteria (Almeida and Almeida, 1999).

The use of the GDS for the detection of depressive symptoms and diagnosis of a depressive episode in AD patients is controversial. Some studies report a loss of validity in patients with dementia (Feher et al., 1992), while others do not (Brown et al., 2007). The previous reports suggests that patients in moderate or severe dementia may not report depressive symptoms accurately. To reduce these biases we selected only patients with mild dementia for the AD group (CDR ≤ 1). Due to participant's low formal education, to ensure the questions comprehension and validity of the report the examiner read the questions aloud. The diagnosis of AD, MCI or NA, was irrespective of the GDS-15 scores.

### Neuropsychological assessment (research protocol)

All participants underwent a comprehensive neuropsychological assessment protocol focused on the assessment of executive functions, memory, language, and visuospatial cognitive domains. The protocol was designed for the assessment of older adults with low formal education level. We detailed these procedures in a previous study conducted in this sample (de Paula et al., 2013b). We have not used these neuropsychological tests were for diagnostic purpose, and they were administered by trained neuropsychologists.

The battery is comprised by the following tests: Digit Span Forward and Backward (Kessels et al., 2008), verbal fluency “fruits,” “animals,” and letter (“S”) (de Paula et al., 2013b), and the Frontal Assessment Battery (Dubois et al., 2000) [Factor *Executive Functions*]; the three components of the Laboratory of Neuropsychological Investigations Naming Test (Malloy-Diniz et al., 2007a) [Factor *Language/Semantic Memory*]; the Brazilian Portuguese Rey Auditory-Verbal Learning Test (Malloy-Diniz et al., 2007b) [Factor *Episodic Memory*]; Token Test components of attention and comprehension (De Renzi and Faglioni, 1978; de Paula et al., 2013b), the Clock Drawing Test (Shulman, 2000); and the Stick Design Test (Baiyewu et al., 2004) [Factor *Visuospatial Abilities*]. Supplementary Table 1 shows the participant's performance in each neuropsychological measures, its clinical cut-offs and maximum possible score. Supplementary Table 2 shows the factor analysis.

### Activities of daily living assessment

Participant's caregivers (usually spouses or other relatives) answered an adapted Activities of Daily Living Scale (ADL) based on the Lawton and Brody (1969), and Katz et al. (1970) indexes of ADL. The scale assess 13 basic and instrumental activities (de Paula et al., 2014). The scale consists of three ADL aspects: Self-Care (Basic ADL), Domestic (Instrumental ADL performed at home), and Complex (Instrumental ADL involving activities outside home, financial management and medication control). Scores on Basic ADL range from 0 to 10, and 0 to 8 on Domestic or Complex ADL; with lower scores indicating greater impairment. Due to data distribution, we categorized the functional performance (impairment × no-impairment) based in dependence of human help (full or partial) to perform at least one ADL. Supplementary Table 1 shows the participant's functional performance in each subscale, while Supplementary Table 3 shows the frequency of responses by each group, stratified by depression status, in each specific ADL.

### Statistical analysis

Prior to this study analysis, we carried out a factor analysis with all neuropsychological measures for data reduction, as previously reported (de Paula et al., 2013b, 2015a). Principal axis factoring and oblique rotation were adopted. We extracted and saved the cognitive factors as standardized (z-Scores) variables based on non-depressed NA performance. The procedures produced four distinct cognitive factors (Executive Functions, Language/Semantic Memory, Episodic Memory, and Visuospatial Abilities), used as dependent variables in the multivariate analysis. All the factors showed adequate factor loadings and internal consistency assessed by the Cronbach's Alpha (all >0.800).

For cognitive data we carried out MANOVA analysis to assess the main effect of group (NA × MCI × AD) and clinically significant depressive symptoms according to GDS cut-off (Depressed × Non-Depressed) on each neuropsychological factor. Age and education were used as covariates as they are significantly correlated with cognitive performance in these subjects (Supplementary Table 4). We analyzed the MANOVA residuals to check if their distribution meet the analysis assumptions. We reported effect sizes by partial eta-squared (η${}_{p}^{2}$). Since neither age nor education correlated with functional measures after controlling for global cognitive functioning in this sample (Supplementary Table 4), we adopted a multinomial logistic regression model to compare the frequency of impairment in each aspect of ADL. Group and depression were added as fixed factors and chi-square tests were adopted for *post-hoc* analysis. Effect sizes for chi-square tests were estimated by the Cramér's phi (φ). *Post-hoc* analysis comparisons were corrected for multiple comparisons.

We performed most of the statistical procedures on the SPSS 20.0. Statistical significance was established at 0.05 (two-tailed). Power analysis for main effects was done in G-Power software (Faul et al., 2007). For MANOVA, our sample size (*n* = 274) has a 99% power to detect a large effect size, 86% power to detect a moderate effect size and only 17% power to detect a small effect size. We computed power analysis for logistic regression based in the effect sizes represented by different odds ratio, as proposed by Chen et al. (2010): 1.68 (small effect size), 3.47 (medium effect size), 6.71 (large effect size). Based on our sample size we have 93% power to detect a small effect size and 99% power to detect a small or large effect size in logistic regression.

## Results

Our final sample consisted of 274 participants: 62 NA non-depressed, 34 NA depressed, 63 MCI non-depressed, 22 MCI depressed, 66 AD non-depressed and 27 AD depressed. We did not observe a statistically significant difference in the distribution of depression according to diagnostic groups (χ^2^ = 2.05, *p* = 0.359). Table 2 shows neuropsychological and functional data for each subgroup. Supplementary Tables 1, 2 shows the scores in specific neuropsychological tests and functional status in each ADL. Performance of NA participants were relatively similar to what is expected for older adults with low formal education, according to Brazilian normative studies, but lower than what is expected for young adults (Brucki and Rocha, 2004; Banhato and Nascimento, 2007; Malloy-Diniz et al., 2007b; Aprahamian et al., 2009; Moreira et al., 2011; de Paula et al., 2015b).

**Table 2:** **Participant's scores in GDS-15 and performance in the neuropsychological factors obtained by factor analysis of the research protocol stratified by group and depression**.

**Group**	**Depression**	**Statistic**	**GDS-15**	**Language semantic**	**Episodic memory**	**Visuospatial abilities**	**Executive functions**	**G-ADL self-care**	**G-ADL domestic**	**G-ADL complex**	**G-ADL general**
Normal aging (*N* = 96)	Non-depressed (*N* = 62)	*M*	1.81	0.00	0.00	0.00	0.00	9.92	7.92	7.85	25.69
		*SD*	1.30	1.00	1.00	1.00	1.00	0.38	0.33	0.51	0.93
		Min-Max	0 to 5	−3.54 to 1.39	−1.60 to 1.14	−2.62 to 1.70	−2.70 to 1.63	8 to 10	6 to 8	5 to 8	20 to 26
	Depressed (*N* = 34)	*M*	8.94	−0.99	−0.79	−0.87	−1.63	9.97	7.24	7.00	24.21
		*SD*	2.80	1.26	0.77	0.99	1.25	0.17	1.28	1.92	3.02
		Min-Max	6 to 15	−3.54 to 1.13	−2.72 to 0.86	−2.46 to 1.12	−5.80 to 0.90	9 to 10	4 to 8	1 to 8	15 to 26
Mild cognitive impairment (*N* = 85)	Non-depressed (*N* = 63)	*M*	2.06	−0.75	−1.30	−0.87	−1.19	10.00	7.41	6.94	24.35
		*SD*	1.50	1.05	0.73	1.09	1.05	0.00	1.25	1.45	2.28
		Min-Max	0 to 5	−3.52 to 1.05	−2.74 to 0.70	−2.70 to 2.07	−3.70 to 1.00	10 to 10	2 to 8	2 to 8	16 to 26
	Depressed (*N* = 22)	*M*	5.45	−0.76	−1.01	−0.76	−1.28	9.95	7.41	6.82	24.18
		*SD*	4.08	0.67	0.66	0.90	1.09	0.21	1.05	1.56	2.56
		Min-Max	6 to 13	−2.05 to 0.40	−2.19 to 0.35	−2.34 to 0.80	−3.30 to 0.30	9 to 10	5 to 8	3 to 8	18 to 26
Mild Alzheimer's dementia (*N* = 93)	Non-depressed (*N* = 66)	*M*	2.05	−1.74	−1.81	−1.62	−2.44	9.86	5.76	4.53	20.15
		*SD*	1.39	1.22	0.64	1.06	1.04	0.52	2.11	2.53	4.00
		Min-Max	0 to 5	−3.54 to 0.78	−2.78 to 0.39	−2.70 to 2.58	−5.20 to 0.40	7 to 10	0 to 8	0 to 8	10 to 26
	Depressed (*N* = 27)	*M*	8.19	−1.55	−1.64	−1.12	−1.79	9.59	5.70	3.93	19.22
		*SD*	2.04	1.01	0.66	0.94	1.09	1.39	2.40	2.67	5.49
		Min-Max	6 to 14	−2.85 to 0.40	−3.00 to 0.00	−2.60 to 1.09	−3.30 to 1.50	3 to 10	0 to 8	0 to 8	9 to 26

*GDS-15, Geriatric Depression Scale 15-items; M, Mean; SD, Standard deviation; Min, Minimum; Max, maximum*.

Since, in previous studies we explored the effect of group in the neuropsychological and functional measures (de Paula et al., 2013b, 2014), we focused in the interaction between group and depression in cognitive and functional measures. We found significant interactions for all measures (Table 3).

**Table 3:** **Interactions between group (NA × MCI × AD) and depression (present × absent) in cognitive factors and functional measures**.

**Cognitive and functional measures**	**Group × Depression**	
	***F*/χ^2^**	***p***
Language/Semantic memory^a^	4.61	0.011
Episodic memory^a^	12.05	< 0.001
Executive functions^a^	20.79	0.002
Visuospatial abilities^a^	6.29	< 0.001
Self-care ADL^b^	3.00	0.223
Domestic ADL^b^	8.31	0.016
Complex ADL^b^	6.55	0.038
General ADL^b^	8.84	0.012

*NA, Normal aging; MCI, Mild Cognitive Impairment; AD, Alzheimer's disease dementia; ADL, Activities of Daily Living*.

a *MANOVA (group × depression) covariating age and education for cognitive factors*.

b *Logistic regression (group × depression) for functional measures*.

**Figure 2:**
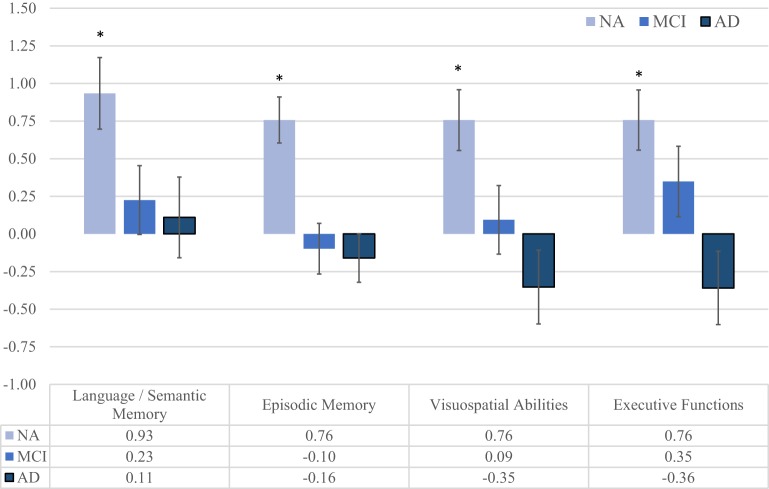
**Differences between the estimated marginal means (with age and education as covariates) on neuropsychological measures of depressed and non-depressed participants in each of the three groups**. When we compared depressed and non-depressed participants in each group, we found significant differences only in NA participants. In this group, participants with clinically significant symptoms of depression showed lower performance in Executive Functions (*p* < 0.001, η_*p*_^2^ = 0.37), Episodic Memory (*p* < 0.001, η_*p*_^2^ = 0.21), Language/Semantic Memory (*p* < 0.001, $\eta_{p}^{2}$ = 0.14), and Visuospatial Abilities (*p* = 0.001, η_*p*_^2^ = 0.12). Effect sizes from MCI and AD patients ranged from ^*^p < 0.01 to 0.03.

For cognitive measures, interactions were significant between depressed and non-depressed NA participants, and the largest effect size occurred in executive functions (η_*p*_^2^ = 0.37), followed by episodic memory (η_*p*_^2^ = 0.21), language/semantic memory (η_*p*_^2^ = 0.14) and visuospatial abilities (η_*p*_^2^ = 0.12). However, in MCI and AD participant depression was not associated with cognitive performance (non-significant effect sizes ranging from η_*p*_^2^ < 0.01 to η_*p*_^2^ = 0.03). Differences remained significant after multiple comparisons correction. These data is show in Table 4 and Figure 2.

**Table 4:** **Main effect of depression in cognitive functions and group comparison's after covariating age and education**.

	**Non-depressed^a^**	**Depressed^a^**	***p***	**η*_*p*_*^2^**
**NORMAL AGING**				
Language/Semantic memory	−0.01 (0.14)	−0.94 (0.19)	< 0.001	0.14
Episodic memory	−0.01 (0.09)	−0.77 (0.12)	< 0.001	0.21
Visuospatial abilities	−0.05 (0.12)	−0.77 (0.16)	0.001	0.12
Executive functions	−0.06 (012)	−1.52 (0.16)	< 0.001	0.37
**MILD COGNITIVE IMPAIRMENT**				
Language/Semantic memory	−0.70 (0.11)	−0.92 (0.20)	0.329	0.01
Episodic memory	−1.25 (0.08)	−1.15 (0.14)	0.561	0.01
Visuospatial abilities	−0.82 (0.11)	−0.91 (0.20)	0.683	0.01
Executive functions	−1.12 (0.12)	−1.47 (0.20)	0.140	0.03
**ALZHEIMER's DISEASE DEMENTIA**				
Language/Semantic memory	−1.66 (0.14)	−1.77 (0.22)	0.682	0.01
Episodic memory	−1.81 (0.08)	−1.65 (0.13)	0.324	0.01
Visuospatial abilities	−1.58 (0.13)	−1.22 (0.20)	0.154	0.02
Executive functions	−2.35 (0.13)	−1.99 (0.20)	0.146	0.02

a *Estimated marginal mean (standard error)*.

**Figure 3:**
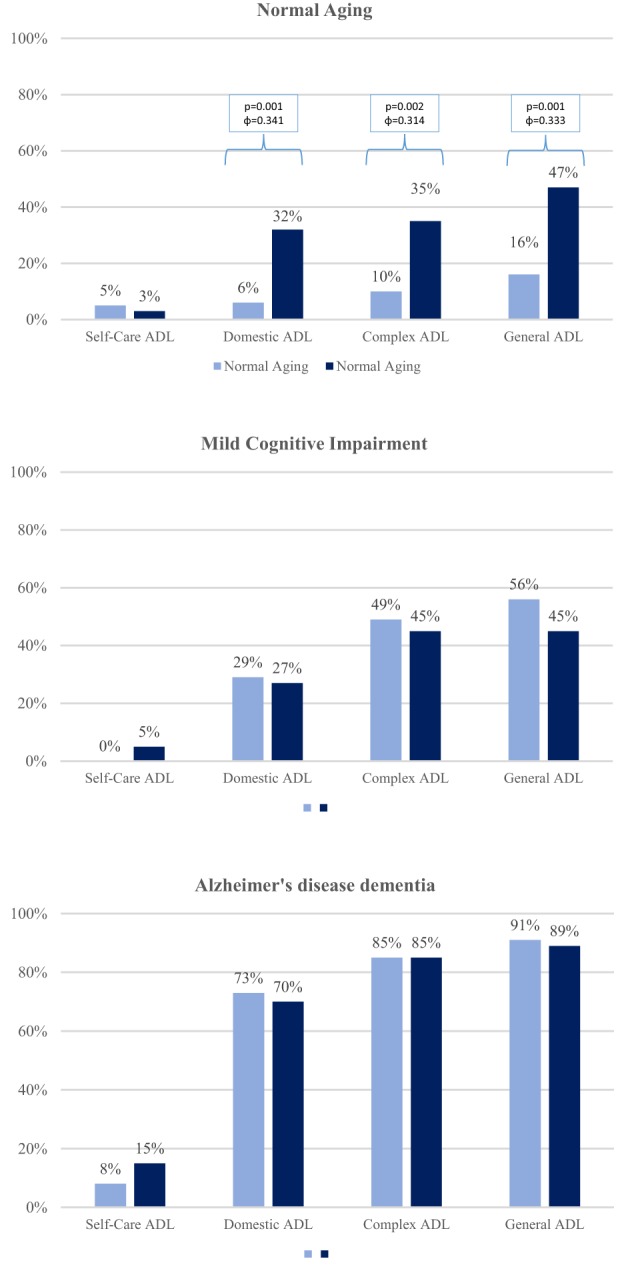
**Associations between the frequency deficits in different types of ADL and the presence of clinically significant symptoms of depression in each group**. We found significant differences in frequency of impairment (partial or complete) of at least one ADL and the presence of clinically significant symptoms of depression only in the NA group. Impairment in instrumental-domestic ADL (*p* = 0.001, φ = 0.341), instrumental-complex ADL (*p* = 0.002, φ = 0.314), and ADL general (*p* = 0.001, φ = 0.333) were more frequent in participants with clinically significant depressive Symptoms. The association between depression and ADL was found in MCI or AD participants (effect sizes ranged from 0.004 to 0.185).

The interaction for group × depression in self-care ADL was not significant (*p* = 0.223). For instrumental-domestic activities, we found a significant difference in the NA group (φ = 0.341), where difficulties were more frequent in depressed (32%) than non-depressed (6%) participants. The same pattern occurred for instrumental-complex activities (φ = 0.314), where depressed patients (35%) showed more difficulties than non-depressed participants (10%). Considering all the 13 activities, a significant difference also occurred in the NA group (φ = 0.333), where depressed participants were more likely to show impairment in at least one activity (47%) than non-depressed participants (16%). However, for both MCI and AD participants, no significant differences were found on instrumental-domestic, instrumental-complex, or in the general index of ADL (non-significant effect sizes ranging from φ = 0.004 to φ = 0.185). Differences remained significant after multiple comparisons correction. Table 5 and Figure 3 show these analyses.

**Table 5:** **Frequency of impairment in at least one activity of daily living in each group and group comparison's (chi-square test) between depressed and non-depressed participants**.

		**NA**		**MCI**		**AD**	
	**Impairment^a^**	**ND**	**D**	**ND**	**D**	**ND**	**D**
Self-care ADL	No	95%	97%	100%	95%	92%	85%
	Yes	5%	3%	0%	5%	8%	15%
		χ^2^ = 0.20		χ^2^ = 2.89		χ^2^ = 1.15	
		*p* = 0.656		*p* = 0.259		*p* = 0.284	
		φ = 0.045		φ = 0.185		φ = 0.024	
Domestic ADL	No	94%	68%	71%	73%	27%	30%
	Yes	6%	32%	29%	27%	73%	70%
		χ^2^ = 11.17		χ^2^ = 0.01		χ^2^ = 0.05	
		*p* = 0.001		*p* = 0.907		*p* = 0.818	
		φ = 0.341		φ = 0.013		φ = 0.185	
Complex ADL	No	90%	65%	51%	55%	15%	15%
	Yes	10%	35%	49%	45%	85%	85%
		χ^2^ = 9.46		χ^2^ = 0.09		χ^2^ = 0.01	
		*p* = 0.002		*p* = 0.762		*p* = 0.967	
		φ = 0.314		φ = 0.033		φ = 0.004	
General ADL	No	84%	53%	44%	55%	9%	11%
	Yes	16%	47%	56%	45%	91%	89%
		χ^2^ = 10.64		χ^2^ = 0.67		χ^2^ = 0.09	
		*p* = 0.001		*p* = 0.414		*p* = 0.765	
		φ = 0.333		φ = 0.089		φ = 0.031	

*NA, Normal Aging; MCI, Mild Cognitive Impairment; AD, Alzheimer's disease dementia; D, Depressed; ND, Non-Depressed; ADL, Activities of daily living*.

a *Requires partial need of human help to perform at least one ADL of each group*.

## Discussion

The present results suggest that the association of depressive symptoms with cognitive and functional performance is dependent on the degree of global impairment across the NA-MCI-AD continuum. The effect of depression on cognitive functions such as episodic memory, language, executive functions, and visuospatial abilities, was more intense in the NA group. Depressive symptoms in subjects with MCI or AD had no significant effect on cognitive performance, with very low effect sizes. We found an association between depression and functional measures only in the NA group, and this effect occurred in instrumental-domestic, instrumental-complex, and general-ADL. In this sense, our results suggest that depression not be strongly associated with a worse cognitive and functional phenotype in patients with MCI or AD.

The effect of depressive symptoms was more pronounced in executive function in comparison to the other cognitive domains. Executive dysfunction impairment is an important feature of late-life depression and is associated with negative outcomes such as suicide behavior, development of neuropsychiatric symptoms, and higher risk of dementia (Moreira et al., 2012; Richard-Devantoy et al., 2012; Diniz et al., 2013). As the integrity of the fronto-striatal circuitry and dopaminergic neurotransmission are main correlates of executive functions, we can hypothesize that these neurobiological correlates are particularly vulnerable to the deleterious effects of depression (Fitzgerald et al., 2008; Malloy-Diniz et al., 2013). In this sense, cognitive interventions, neuromodulation, psychotherapy, or pharmacological treatment may restore or reduce the intensity of both cognitive deficits and psychiatric symptoms (Lenze et al., 2014; Mackin et al., 2014; Diamond et al., 2015). However, this is a complex relationship, since improvement of depressive symptoms may not necessarily lead to remission in cognitive deficits (Nebes et al., 2003; Bhalla et al., 2006) or improvement in cognitive functioning may not reduce symptoms of depression (McDermott and Gray, 2012).

Cognitive impairment in depression has been associated to distinct neurobiological changes. Depression is associated with the hypothalamic-pituitary-adrenal axis dysfunction, a factor related to higher secretion of glucocorticoids (Butters et al., 2008). This mechanism relates to hippocampal atrophy and may be a neurobiological causal factor to the episodic memory impairment in depressed subjects (Panza et al., 2010; Naismith et al., 2012). Another important mechanism with may explain the current findings is the increase of vascular burden, especially white matter lesions, found in depressed subjects (Tham et al., 2011). These lesions may reflect on the overall brain functioning by ischemia or the disruption of frontostriatal connections, aspects closely related to the executive functions and processing speed (Royall et al., 2002). A third pathway relates to amyloid burden: late-life depression is associated to an increase of β-amyloid, a factor associated with the severity of cognitive impairment and overall severity of depression (Piccinni et al., 2013).

However, similar changes were reported in MCI subjects and patients with AD (Forlenza et al., 2010; Panza et al., 2010). Therefore, the presence of these neurobiological changes in subjects with depressive symptoms and MCI/AD seems not to confer a synergic or an additive effect on cognitive performance in older adults. On the other hand, the emergence of neurodegenerative changes in the MCI/AD continuum may reduce the impact of depression-related neurobiological changes on cognitive performance. Additional studies, including neuroimaging and biological markers, are necessary to disentangle the mechanisms by which depressive symptoms moderate cognitive performance in these subjects.

We evaluated the impact of depressive symptoms over a broad range of ADLs. Depressive symptoms were associated with worse performance only on instrumental-domestic or instrumental-complex ADL. This pattern of association might be explained by the complex nature of the tasks by itself, which my demand more pronounced involvement motivation, emotional regulation, and cognitive control. In addition, the effects of depression in ADL seems weaker than its impact in cognitive functioning. Our results are in line with recent reports of the literature (Reppermund et al., 2011; Park et al., 2013; de Paula et al., 2015a). Previous studies also showed no significant impact of depression in ADLs. Methodological differences between studies may help to explain the effects results, in particular sample setting (population-based, memory clinic and nursing homes) and different strategies and scales to assess ADLs (objective assessment, informant-based assessment; Reppermund et al., 2011). Given the importance of this topic, additional studies are necessary to address the impact of late-life depression in the performance on ADLs.

The present study should be viewed in light of some limitations, which hinders its generalization to other populations. First, we used the GDS-15 to evaluate depressive symptoms and we did not carry out a structured psychiatric interview to confirm the presence of a depressive episode. Despite the fact that GDS-15 is widely used in clinical and research settings, there is criticism about the use of the GDS-15 on the assessment of depressive symptoms in AD and other dementias. Therefore, we might have misidentified cases of depression in AD group, what may have influenced the present results. The cross-sectional design and the recruitment of participants among individuals referred to a cognitive evaluation due to memory complaints also limit the generalization of the present results. On the other hand, cognitive performance was evaluated by a comprehensive neuropsychological protocol, which was not used to define each subject diagnosis. This is a major strength of our study and reduces the risk of circularity and bias in the present analyses.

In conclusion, the present study reanalyzed a previously published dataset and showed a significant association of depressive symptoms on multiples domains of cognition only in older subjects with no evidence of global cognitive impairment. In addition, the effect of depressive symptoms on functional performance was restricted to domestic and complex ADLs. Additional studies, with a prospective design and with population-based samples are necessary to replicate the present findings.

## Funding

This work was supported by the following grants: APQ-01972/12-10, APQ-02755-10, APQ-04706-10, CBB-APQ-00075-09 from FAPEMIG, and 573646/2008-2 from CNPq. The funders had no role in study design, data collection, analysis, decision to publish, or preparation of the manuscript.

### Conflict of interest statement

The authors declare that the research was conducted in the absence of any commercial or financial relationships that could be construed as a potential conflict of interest. The reviewer, Maria Semkovska, and the handling Editor declared their shared affiliation, and the handling Editor states that the process nevertheless met the standards of a fair and objective review.
