# Supplementary material for: A Reanalysis of Cognitive-Functional Performance in Older Adults: Investigating the Interaction Between Normal Aging, Mild Cognitive Impairment, Mild Alzheimer's Disease Dementia, and Depression
Source: Front Psychol. 2016 Jan 26;6:2061. doi: 10.3389/fpsyg.2015.02061 (PMC4727063; doi:10.3389/fpsyg.2015.02061)
Supplement: Supplementary file 1 [file Table1.docx]

Supplementary Table 1: Raw scores according to group and presence of depression in neuropsychological and functional measures

|  |  |  | Normal Aging | | | | | | Mild Cognitive Impairment | | | | | | Mild Alzheimer’s dementia | | | | | |
| --- | --- | --- | --- | --- | --- | --- | --- | --- | --- | --- | --- | --- | --- | --- | --- | --- | --- | --- | --- | --- |
|  |  |  | Non-Depressed | | | Depressed | | | Non-Depressed | | | Depressed | | | Non-Depressed | | | Depressed | | |
|  | Max | Cut-off | M | SD | M-M | M | SD | M-M | M | SD | M-M | M | SD | M-M | M | SD | M-M | M-M | SD | M-M |
| Age | - | - | 73.94 | 7.84 | 60-88 | 70.21 | 7.12 | 60-84 | 74.46 | 8.47 | 60-95 | 69.50 | 7.45 | 60-88 | 75.91 | 6.05 | 60-88 | 71.30 | 7.03 | 60-86 |
| Education | - | - | 5.89 | 4.38 | 0-19 | 4.00 | 3.89 | 0-15 | 4.51 | 4.08 | 0-17 | 5.27 | 3.81 | 0-15 | 4.42 | 3.14 | 0-13 | 5.78 | 4.04 | 0-15 |
| GDS-15 | 15 | 5 | 1.81 | 1.30 | 0-5 | 8.94 | 2.80 | 2-15 | 2.06 | 1.50 | 0-5 | 5.45 | 4.08 | 0-13 | 2.05 | 1.39 | 0-5 | 8.19 | 2.04 | 6-14 |
| Mini-Mental State Exam | 30 | 22 | 27.00 | 3.75 | 16-30 | 22.85 | 3.62 | 16-29 | 23.52 | 3.73 | 16-30 | 23.50 | 3.36 | 16-29 | 19.91 | 4.07 | 12-28 | 22.26 | 3.27 | 16-29 |
| Category Fluency (Animals) | - | 12 | 15.23 | 4.58 | 7-28 | 11.74 | 4.08 | 1-22 | 11.43 | 3.42 | 6-20 | 10.32 | 3.23 | 4-17 | 7.79 | 3.20 | 2-14 | 10.19 | 4.67 | 4-25 |
| Category Fluency (Fruits) | - | 11 | 13.15 | 3.78 | 5-22 | 9.79 | 3.27 | 3-16 | 9.43 | 2.50 | 3-15 | 9.18 | 1.94 | 6-14 | 7.74 | 2.80 | 2-15 | 8.11 | 2.52 | 3-14 |
| Letter Fluency ("S") | - | 10 | 11.90 | 3.75 | 4-23 | 6.50 | 2.89 | 0-14 | 8.95 | 3.84 | 0-17 | 9.73 | 4.46 | 1-18 | 7.14 | 3.17 | 2-20 | 7.04 | 4.10 | 1-22 |
| Digit Span Forward | 144 | 27 | 38.74 | 19.22 | 12-88 | 25.47 | 11.75 | 5-54 | 36.13 | 14.84 | 9-77 | 29.77 | 10.50 | 20-54 | 27.64 | 13.04 | 6-77 | 32.00 | 18.17 | 12-88 |
| Digit Span Backward | 112 | 11 | 18.10 | 10.48 | 4-54 | 11.06 | 7.11 | 4-36 | 12.65 | 10.64 | 0-60 | 12.09 | 8.84 | 4-35 | 7.73 | 6.37 | 0-42 | 12.04 | 9.01 | 2-42 |
| Frontal Assessment Battery | 18 | 12 | 14.74 | 2.76 | 8-18 | 11.44 | 3.25 | 6-17 | 11.62 | 2.89 | 6-18 | 12.41 | 2.75 | 6-17 | 8.71 | 3.20 | 3-18 | 9.93 | 3.16 | 4-18 |
| TN-LIN (Nouns) | 40 | 26 | 37.81 | 2.47 | 28-40 | 36.15 | 3.02 | 31-40 | 36.81 | 2.67 | 26-40 | 36.86 | 2.01 | 33-40 | 33.45 | 4.21 | 17-40 | 34.15 | 3.16 | 29-40 |
| TN-LIN (Verbs) | 10 | 9 | 9.77 | 0.64 | 7-10 | 9.21 | 1.45 | 4-10 | 9.67 | .76 | 6-10 | 9.64 | .58 | 8-10 | 8.76 | 1.65 | 2-10 | 9.33 | .73 | 8-10 |
| TN-LIN (Professions) | 15 | 14 | 13.87 | 2.18 | 0-15 | 12.47 | 2.97 | 5-15 | 13.02 | 2.04 | 6-15 | 13.27 | 1.16 | 11-15 | 11.12 | 3.41 | 1-15 | 11.67 | 2.30 | 8-15 |
| RAVLT - A1 | 15 | 4 | 4.92 | 1.83 | 2-10 | 4.00 | 1.44 | 1-7 | 3.36 | 1.35 | 1-7 | 3.23 | 1.15 | 1-5 | 2.72 | 1.46 | 0-6 | 3.15 | 1.26 | 2-6 |
| RAVLT - Immediate Recall | 15 | 5 | 7.42 | 3.10 | 2-15 | 4.65 | 2.90 | 0-12 | 3.17 | 2.33 | 0-11 | 4.36 | 2.13 | 0-8 | 2.20 | 1.87 | 0-6 | 2.11 | 2.17 | 0-6 |
| RAVLT - Dealyed Recall | 15 | 5 | 7.69 | 3.34 | 0-15 | 4.47 | 2.96 | 0-12 | 2.99 | 2.39 | 0-9 | 3.82 | 2.84 | 1-10 | 1.85 | 1.87 | 0-7 | 1.93 | 1.75 | 0-7 |
| RAVLT – Recognition | 15 | 5 | 8.90 | 4.30 | -1-15 | 5.72 | 4.00 | -2-13 | -.21 | 6.30 | -15-13 | 3.59 | 6.92 | -9-19 | -1.89 | 6.52 | -20-10 | .00 | 5.49 | -11-8 |
| RAVLT – Total | 75 | 38 | 38.32 | 11.89 | 5-64 | 29.15 | 9.18 | 11-52 | 25.94 | 9.38 | 0-54 | 27.41 | 6.49 | 15-42 | 20.55 | 7.63 | -9-36 | 23.48 | 6.74 | 8-41 |
| Clock Drawing Test | 5 | 3 | 4.10 | 1.18 | 1-5 | 2.47 | 2.18 | 0-5 | 2.77 | 1.68 | 0-5 | 2.36 | 1.92 | 0-5 | 1.70 | 1.58 | 0-5 | 2.78 | 1.42 | 0-5 |
| Stick Design Test | 12 | 11 | 11.92 | .33 | 10-12 | 11.18 | 1.34 | 6-12 | 11.11 | 1.84 | 1-12 | 11.00 | 1.77 | 6-14 | 10.12 | 2.51 | 2-12 | 10.63 | 2.22 | 2-12 |
| Token Test (Attention) | 15 | 15 | 14.82 | .59 | 11-15 | 14.59 | 0.78 | 12-15 | 14.55 | .87 | 11-15 | 14.69 | .55 | 13-15 | 14.22 | 1.18 | 10-15 | 14.58 | .79 | 12-15 |
| Token Test (Comprehension) | 21 | 11 | 16.85 | 2.86 | 7-21 | 14.81 | 3.26 | 8-21 | 14.40 | 3.39 | 5-21 | 15.32 | 2.47 | 11-19 | 12.07 | 4.14 | 4-21 | 14.02 | 3.10 | 7-20 |
| G-ADL - Self-care | 10 | 9 | 9.92 | 0.38 | 8-10 | 9.97 | 0.17 | 9-10 | 10.00 | 0.00 | 10-10 | 9.95 | .21 | 9-10 | 9.86 | .52 | 7-10 | 9.59 | 1.39 | 3-10 |
| G-ADL - Instrumental-Domestic | 8 | 7 | 7.92 | 0.33 | 6-8 | 7.24 | 1.28 | 4-8 | 7.41 | 1.25 | 2-8 | 7.41 | 1.05 | 5-8 | 5.76 | 2.11 | 0-8 | 5.70 | 2.40 | 0-8 |
| G-ADL - Instrumental-Complex | 8 | 6 | 7.85 | 0.51 | 5-8 | 7.00 | 1.92 | 1-8 | 6.94 | 1.45 | 2-8 | 6.82 | 1.56 | 3-8 | 4.53 | 2.53 | 0-8 | 3.93 | 2.67 | 0-8 |
| G-ADL – General | 26 | 23 | 25.69 | 0.93 | 20-26 | 24.21 | 3.02 | 15-26 | 24.35 | 2.28 | 16-26 | 24.18 | 2.56 | 18-26 | 20.15 | 4.00 | 10-26 | 19.22 | 5.49 | 3-26 |

GDS-15: Geriatric Depression Scale 15-items, TN-LIN: Neuropsychological Investigations Laboratory Naming Test, RAVLT: Rey Auditory Verbal Learning Test, G-ADL: General Activities of Daily Living Scale. M: mean, SD: standard deviation, M-M: Minimum-Maximum
