# Supplementary material for: A Reanalysis of Cognitive-Functional Performance in Older Adults: Investigating the Interaction Between Normal Aging, Mild Cognitive Impairment, Mild Alzheimer's Disease Dementia, and Depression
Source: Front Psychol. 2016 Jan 26;6:2061. doi: 10.3389/fpsyg.2015.02061 (PMC4727063; doi:10.3389/fpsyg.2015.02061)
Supplement: Supplementary file 2 [file Table2.DOCX]

Supplementary Table 2: Factor structure of the neuropsychological assessment protocol (published in de Paula et al., 2013)

| Neuropsychological Test | Executive  Functions | Language /  Semantic Memory | Episodic  Memory | Visuospatial  Abilities |
| --- | --- | --- | --- | --- |
| Letter Fluency (S) | **0.649** | 0.099 | -0.286 | -0.070 |
| Category Fluency (Animals) | **0.579** | 0.035 | -0.313 | -0.141 |
| Category Fluency (Fruits) | **0.503** | 0.026 | -0.318 | -0.086 |
| Frontal Assessment Battery | **0.435** | 0.121 | -0.126 | 0.288 |
| Digit Span Forward | **0.426** | -0.018 | 0.064 | 0.115 |
| Digit Span Backward | **0.417** | 0.065 | 0.062 | 0.247 |
| TN-LIN (Professions) | -0.021 | **0.974** | 0.016 | -0.075 |
| TN-LIN (Verbs) | -0.001 | **0.839** | 0.070 | -0.017 |
| TN-LIN (Nouns) | -0.023 | **0.760** | -0.076 | 0.103 |
| RAVLT (IR) | -0.006 | -0.014 | **-0.918** | -0.037 |
| RAVLT (DR) | -0.033 | 0.054 | **-0.910** | -0.030 |
| RAVLT (Total) | 0.070 | 0.011 | **-0.810** | 0.001 |
| RAVLT (A1) | -0.015 | -0.006 | **-0.669** | 0.133 |
| RAVLT (Rec) | 0.003 | 0.028 | **-0.558** | 0.189 |
| Token Test – Comprehension | 0.286 | 0.075 | -0.041 | **0.508** |
| Token Test – Attention | -0.013 | 0.046 | -0.049 | **0.491** |
| Stick Design Test | 0.041 | 0.046 | -0.128 | **0.461** |
| Clock Drawing Test | 0.304 | 0.135 | -0.119 | **0.318** |

RAVLT: Rey Auditory-Verbal Learning Test, IR: Immediate Recall, DR: Delayed Recall, Rec: Recognition, TN-LIN: Laboratory of neuropsychological Investigations Naming Test.

de Paula, J.J., Bertola, L., Ávila, R.T., Moreira, L., Coutinho, G., Moraes, E.N., et al. (2013). Clinical applicability and cutoff values for an unstructured neuropsychological assessment protocol for older adults with low formal education. PLoS. One. 8(9), e73167.
