# Supplementary material for: A Reanalysis of Cognitive-Functional Performance in Older Adults: Investigating the Interaction Between Normal Aging, Mild Cognitive Impairment, Mild Alzheimer's Disease Dementia, and Depression
Source: Front Psychol. 2016 Jan 26;6:2061. doi: 10.3389/fpsyg.2015.02061 (PMC4727063; doi:10.3389/fpsyg.2015.02061)
Supplement: Supplementary file 3 [file Table3.DOCX]

Supplementary Table 3: frequency impairment in each activity of daily living stratified by group (NA, MCI, AD) and depression (D x ND)

|  |  | NA-ND | NA-D | MCI-ND | MCI-D | AD-ND | AD-D |
| --- | --- | --- | --- | --- | --- | --- | --- |
| The patient uses the shower, soap and bush without assistance? | Indpedent | 100% | 100% | 100% | 100% | 95% | 96% |
|  | Partialy dependent | 0% | 0% | 0% | 0% | 3% | 0% |
|  | Dependent | 0% | 0% | 0% | 0% | 2% | 4% |
| The patient gets clothes and gets completely dressed without assistance? | Indpedent | 100% | 97% | 100% | 100% | 98% | 88% |
|  | Partialy dependent | 0% | 3% | 0% | 0% | 2% | 8% |
|  | Dependent | 0% | 0% | 0% | 0% | 0% | 4% |
| The patient goes to the toilet room (…) cleans self after elimination (…) without help? | Indpedent | 100% | 100% | 100% | 100% | 98% | 92% |
|  | Partialy dependent | 0% | 0% | 0% | 0% | 2% | 8% |
|  | Dependent | 0% | 0% | 0% | 0% | 0% | 0% |
| The patient moves in and out of bed as well as in and out of chair without assistance? | Indpedent | 98% | 100% | 100% | 95% | 97% | 96% |
|  | Partialy dependent | 0% | 0% | 0% | 5% | 3% | 4% |
|  | Dependent | 2% | 0% | 0% | 0% | 0% | 0% |
| The patient feeds self without assistance? | Indpedent | 100% | 100% | 100% | 100% | 98% | 96% |
|  | Partialy dependent | 0% | 0% | 0% | 0% | 2% | 4% |
|  | Dependent | 0% | 0% | 0% | 0% | 0% | 0% |
| The patient manages financial matters independently? | Indpedent | 100% | 81% | 80% | 81% | 46% | 35% |
|  | Partialy dependent | 0% | 7% | 16% | 19% | 30% | 30% |
|  | Dependent | 0% | 11% | 3% | 0% | 24% | 35% |
| The patient is able to do simple shopping independently? | Indpedent | 98% | 82% | 80% | 81% | 36% | 35% |
|  | Partialy dependent | 2% | 18% | 18% | 10% | 33% | 26% |
|  | Dependent | 0% | 0% | 2% | 10% | 31% | 39% |
| The patient is responsible for taking medication in correct dosages at correct time? | Indpedent | 98% | 79% | 70% | 67% | 31% | 39% |
|  | Partialy dependent | 0% | 11% | 23% | 24% | 36% | 17% |
|  | Dependent | 2% | 11% | 7% | 10% | 33% | 43% |
| The patient is able to go out alone to distant locations using transport? | Indpedent | 98% | 79% | 80% | 71% | 49% | 26% |
|  | Partialy dependent | 2% | 14% | 16% | 29% | 31% | 48% |
|  | Dependent | 0% | 7% | 3% | 0% | 20% | 26% |
| The patient is able to do small domestic chores? | Indpedent | 100% | 82% | 89% | 90% | 64% | 70% |
|  | Partialy dependent | 0% | 18% | 10% | 10% | 22% | 17% |
|  | Dependent | 0% | 0% | 2% | 0% | 15% | 13% |
| The patient is able to do personal laundry completely? | Indpedent | 98% | 82% | 87% | 86% | 64% | 61% |
|  | Partialy dependent | 2% | 14% | 10% | 14% | 22% | 17% |
|  | Dependent | 0% | 4% | 3% | 0% | 15% | 22% |
| The patient is able to operates telephone independently (receive and make calls)? | Indpedent | 100% | 86% | 88% | 76% | 65% | 57% |
|  | Partialy dependent | 0% | 14% | 10% | 24% | 24% | 26% |
|  | Dependent | 0% | 0% | 2% | 0% | 11% | 17% |
| The patient is able to plan and prepare meals independently? | Indpedent | 100% | 79% | 89% | 86% | 51% | 61% |
|  | Partialy dependent | 0% | 18% | 8% | 14% | 31% | 26% |
|  | Dependent | 0% | 4% | 3% | 0% | 18% | 13% |

NA: Normal aging, MCI: Mild Cognitive Impairment, AD: Alzheimer’s disease dementia, ND: non-depressed, D: depressed
