# Supplementary material for: A Reanalysis of Cognitive-Functional Performance in Older Adults: Investigating the Interaction Between Normal Aging, Mild Cognitive Impairment, Mild Alzheimer's Disease Dementia, and Depression
Source: Front Psychol. 2016 Jan 26;6:2061. doi: 10.3389/fpsyg.2015.02061 (PMC4727063; doi:10.3389/fpsyg.2015.02061)
Supplement: Supplementary file 4 [file Table4.DOCX]

Supplementary table 4: Partial correlations between age, education, activities of daily living and cognitive measures

|  |  |  | 1 | 2 | 3 | 4 | 5 | 6 | 7 | 8 | 9 | 10 |
| --- | --- | --- | --- | --- | --- | --- | --- | --- | --- | --- | --- | --- |
| 1 | Age | *r* | 1 | -0.117 | -0.032 | -0.118 | -0.104 | -0.123 | -0.168 | -0.177 | -0.145 | -0.206 |
|  |  | *p* | . | 0.054 | 0.593 | 0.051 | 0.086 | 0.042 | 0.005 | 0.003 | 0.017 | 0.001 |
| 2 | Education | *r* |  | 1 | -0.062 | -0.002 | 0.022 | 0.002 | 0.329 | 0.240 | 0.439 | 0.471 |
|  |  | *p* |  | . | 0.311 | 0.967 | 0.714 | 0.979 | <0.001 | <0.001 | <0.001 | <0.001 |
| 3 | Self-care AD | *r* |  |  | 1 | 0.266 | 0.237 | 0.434 | -0.029 | -0.059 | -0.069 | 0.045 |
|  |  | *p* |  |  | . | <0.001 | <0.001 | <0.001 | 0.630 | 0.328 | 0.253 | 0.460 |
| 4 | Domestic ADL | *r* |  |  |  | 1 | 0.550 | 0.846 | 0.148 | 0.109 | 0.092 | 0.250 |
|  |  | *p* |  |  |  | . | <0.001 | <0.001 | 0.014 | 0.072 | 0.13 | <0.001 |
| 5 | Complex ADL | *r* |  |  |  |  | 1 | 0.890 | 0.255 | 0.168 | 0.098 | 0.246 |
|  |  | *p* |  |  |  |  | . | <0.001 | <0.001 | 0.005 | 0.106 | <0.001 |
| 6 | General ADL | *r* |  |  |  |  |  | 1 | 0.216 | 0.141 | 0.090 | 0.271 |
|  |  | *p* |  |  |  |  |  | . | <0.001 | 0.020 | 0.139 | <0.001 |
| 7 | Language / Semantic Memory | *r* |  |  |  |  |  |  | 1 | 0.287 | 0.527 | 0.500 |
|  |  | *p* |  |  |  |  |  |  | . | <0.001 | <0.001 | <0.001 |
| 8 | Episodic Memory | *r* |  |  |  |  |  |  |  | 1 | 0.277 | 0.394 |
|  |  | *p* |  |  |  |  |  |  |  | . | <0.001 | <0.001 |
| 9 | Visuospatial Abilities | *r* |  |  |  |  |  |  |  |  | 1 | 0.607 |
|  |  | *p* |  |  |  |  |  |  |  |  | . | <0.001 |
| 10 | Executive Functions | *r* |  |  |  |  |  |  |  |  |  | 1 |
|  |  | *p* |  |  |  |  |  |  |  |  |  | . |

ADL: Activities of daily living scale. Correlations controlled by cognitive status (normal aging x mild cognitive impairment x Alzheimer’s dementia).
